# Inflammatory peptides derived from adipose tissue

**DOI:** 10.1186/1742-4933-2-1

**Published:** 2005-01-21

**Authors:** Eric Rudin, Nir Barzilai

**Affiliations:** 1 Institute for Aging Research, Diabetes Research and Training Center, Department of Medicine, Albert Einstein College of Medicine, Bronx, New York, USA

## Abstract

The low-grade inflammation seen with aging is noted particularly in subjects with the metabolic syndrome of aging. Insulin resistance, obesity/abdominal obesity, and risks for many age-related diseases characterize this common syndrome. It is becoming clear  that this increased adipose tissue is not simply a  reservoir for excess nutrients, but rather an active  and dynamic organ capable of expressing several  cytokines and other fat-derived peptides (FDP). Some, but not all, FDP may have a role in development of the metabolic syndrome but there is no evidence that these FDP are causing inflammation directly. We suggest that high levels of inflammatory peptides are markers for obesity/abdominal obesity seen with aging, but some may not necessarily have a causative role in the development of inflammation.

## 

Because bone marrow and adipocytes are derived from the same stem cells, it is not surprising to find so many inflammatory peptides expressed in fat tissue. Many of these are classical inflammatory peptides derived from components of the adipose tissue and all are known also as fat derived peptides (FDP). With aging, there is a linear accumulation of adipose cells and percent of body fat increases. This increased body fat is characterized by increased visceral adiposity [1] and occurs despite the decreased subcutaneous fat and progressive sarcopenia typical of aging [2]. Visceral adiposity has been associated with greater risks for age-related diseases [3]. In addition, fat infiltration typical of aging occurs in many organs including liver and bone marrow. As adipose tissue accumulates throughout the body and in other organs, it is possible that this hyperplastic adipose tissue over expresses FDP.

The metabolic syndrome is a common disorder consisting of a cluster of abnormalities including insulin resistance, dyslipidemia, and hypercoagulability and is associated with increased risk for cancer, Alzheimer's disease, type-II diabetes and atherosclerosis [4]. It is also associated with increased fat mass and increased inflammatory peptides. The obesity epidemic of the rapidly growing aging population makes understanding underlying relationship between adiposity, chronic inflammation and the metabolic syndrome essential.

Increased inflammatory peptides are being studied as possible modifiable markers of the increased risk predictors of disease and possibly the underlying link between obesity and the poor clinical outcomes seen with the metabolic syndrome. More specifically, C-reactive protein (CRP) is the most well established inflammatory cytokine in the clinical setting but there are other inflammatory cytokines including IL-6, leptin, TNF-*α*, and other (non-cytokine) FDP, such as PAI-1, adiponectin and resistin, which may play a role in the pathogenesis, and/or serve as markers of risk in the metabolic syndrome. The fact that many of these peptides are derived from adipose tissue leads us to the question of whether adipose tissue itself is the underlying pathophysiological link between obesity and the poor clinical outcomes associated with the metabolic syndrome. We will provide a brief overview of some of the peptides associated with the metabolic syndrome.

## Cytokines with a potential role in the metabolic syndrome

*TNFα*, leptin, and IL-6 are examples for cytokines that may have a role in the metabolic syndrome. TNF*α*, previously known as lymphotoxin and cachetin, is believed to be involved in the wasting that occurs during acute and chronic illness and malignancy. In the basal state TNF*α *is directly proportional to fat mass and has been shown to be involved in the development of insulin resistance [5]. In-vitro studies have demonstrated that TNF*α *decreases the insulin receptor tyrosine phosphorylation, and down regulates several steps in the insulin signaling pathway [6-9] while neutralizing agents for TNF*α *have been shown to improve insulin resistance. [10] Thus, *TNFα is not only a classical cytokine but may be causal in the insulin resistance of the metabolic syndrome of aging*.

*Leptin *is a peptide derived from adipose tissue and like other cytokines acts through a cytokine receptor. It is expressed and secreted in direct proportion to fat mass. Leptin exerts is effect predominantly through receptors in the hypothalamus but it may also have peripheral actions [5]. Leptin serves as a marker of energy sufficiency by rapidly decreasing during starvation and weight loss. [11] With obesity, leptin levels are increased in proportion to fat mass, but its activity to decrease appetite seems reduced. *Leptin appears to have an important role in energy regulation but no apparent role in development of inflammation*.

*IL-6 *is another cytokine derived from adipose tissue. Its expression and circulating levels correlate directly with obesity, and weight loss will lower circulating levels. Elevation of circulating IL6 is a predictor of the development of cardiovascular disease and diabetes [12]. Infusion of IL6 results in hyperlipidemia, hyperglycemia and insulin resistance in experimental models. [13] Additionally, IL6 decreases the expression adiponectin, an 'anti-diabetic' cytokine. [14]*IL-6 plays a role in the development of insulin resistance and may directly cause induction of CRP*.

Other inflammatory cytokines such as *IL-1*, IL-8, IL18, Serum Amyloid A, have been shown to be increased with obesity and may have a yet undetermined role in the syndrome. These cytokines are other examples of inflammatory markers which do not have a clear role in the causation of systemic inflammation.

## Non-cytokine Fat Derived Peptides with a role in the metabolic syndrome

*Adiponectin *is highly expressed in adipose tissue, and is the one non-cytokine FDP that is protective from inflammation. Unlike most FDP, circulating levels are inversely proportional to obesity and therefore tend to be low in obesity. Adiponectin levels increase with weight loss and with use of insulin sensitizing drugs. [15] Adiponectin administration has been shown to improve insulin sensitivity. [16] Low levels of adiponectin have been linked to inflammatory arthrosclerosis in humans.[17] Animal models have shown that low adiponectin levels increase smooth muscle proliferation in response to injury, increase free fatty acids levels and cause insulin resistance.[18] The pro-diabetic and pro-atherogenic effects of low adiponectin levels seen in the metabolic syndrome provide a link between inflammation and obesity.

*Plasminogen activator inhibitor type-1 (PAI-1) *is the primary inhibitor of fibrinolysis and is highly expressed in adipose tissue. Levels of PAI-1 are elevated in acute conditions such as deep venous thrombosis, and chronic conditions such as obesity, the metabolic syndrome of aging and diabetes. PAI-1 levels are correlated with adiposity and significantly overexpressed in the adipose tissue of obese compared to lean animals. [19] Levels are decreased by weight loss and drugs that improve insulin sensitivity [20]. The relationship of PAI-1 to obesity provides a potential link between the metabolic syndrome and hypercoagulabilty.

*Angiotesinogen (AGT) *is a peptide that is produced in the liver and in adipose tissue. The strong correlation between obesity and hypertension implies that adipose tissue may play a role in blood pressure regulation and in fact there is a correlation between circulating AGT levels and obesity/hypertension [21]. Animal studies have shown that overexpression of AGT results in hypertension while under expression of AGT results in decreased blood pressures [22].

*Resistin *is a peptide which is elevated in obesity and appears to play a role in glucose homeostasis in rodents. In experimental models, resistin induces hepatic insulin resistance while anti-resistin antibodies have the opposite effect [23]. In humans, the role of resistin is less clear and it is not known what role it has glucose homeostasis or whether it directly relates to adipose tissue mass. The role of resistin in pathogenesis of inflammation is also unclear.

## Markers of inflammation or markers of obesity?

Low grade inflammation is a predominant feature in the metabolic syndrome of aging and seems to be linked to the development of diabetes and poor vascular outcomes. We have briefly named several cytokines and other FDP that are generally increased with fat mass. Although many of these FDP have a role in metabolic homeostasis, many seem to lack distinct role in inflammatory pathogenesis. While many FDP have roles in *vivo *metabolism, we suggest that some levels of cytokines are increased because of the hyperplastic characteristic of adipose tissue, and their levels are better serve as marker of adipose tissue hypertrophy, rather than having a causal role in aging. Thus, whether aging is inflammatory state or whether it is a state associated with increased inflammatory marker is subject for further studies.
